# Effects of Normal Reference Range of Phosphorus and Corresponding PTH on Endothelial Function in CKD Patients

**DOI:** 10.3389/fmed.2022.935977

**Published:** 2022-07-12

**Authors:** Shina Lee, Seung-Jung Kim

**Affiliations:** Department of Internal Medicine, School of Medicine, Ewha Womans University, Seoul, South Korea

**Keywords:** phosphorus, fibroblast growth factor 23, parathyroid hormone, endothelial function, chronic kidney disease

## Abstract

**Introduction:**

Endothelial dysfunction commonly occurs in chronic kidney disease (CKD) patients and increases the risk for cardiovascular disease. Among CKD patients, biomarkers involved in the pathogenesis of CKD-mineral bone disorder (CKD-MBD), such as phosphorus, parathyroid hormone, and fibroblast growth factor 23, are associated with endothelial dysfunction. We investigated whether these biomarkers induce endothelial dysfunction in CKD patients with normal phosphorus levels.

**Methods:**

This cross-sectional study examined CKD patients with normal phosphorus levels; patients with an estimated glomerular filtration rate (eGFR) <15 or who were under dialysis were excluded. Iontophoresis with laser doppler flowmetry (ILDF) and peripheral arterial tonometry were performed to assess endothelial function in 85 patients. Pearson's correlation coefficient, multiple regression, and mediation analyses were performed to examine the association between CKD-MBD biomarkers and endothelial dysfunction.

**Results:**

Endothelial dysfunction was observed in all subjects with a low response to ILDF and 27% of subjects according to peripheral arterial tonometry. Acetylcholine (Ach)-induced ILDF was significantly associated with eGFR (*r* = 0.22, *P* = 0.04), intact parathyroid hormone (iPTH; *r* = −0.46, *P* < 0.01), and VCAM-1 (*r* = −0.36, *P* < 0.01). The reactive hyperemia index (RHI) was significantly related to phosphorus levels (*r* = 0.32, *P* < 0.01) and iPTH (*r* = −0.39, *P* = 0.02). After adjusting for eGFR, iPTH and VCAM-1 remained independent factors for predicting endothelial dysfunction measured using Ach-induced ILDF. In addition, iPTH and phosphorus levels were independent predictors for endothelial dysfunction measured using RHI in the eGFR-adjusted model. Mediation analyses showed that the individual indirect effects of iPTH were significantly affected ILDF and RHI.

**Conclusion:**

Serum levels of phosphorus and iPTH are associated with endothelial dysfunction, even in CKD patients with normal phosphorus levels.

## Introduction

Endothelial dysfunction is a multifactorial and complex disorder that contributes to the development of cardiovascular disease, and it is the leading cause of death in chronic kidney disease (CKD) patients (1). Research on the development and progression of endothelial dysfunction is currently lacking, and more studies are needed to improve cardiovascular outcomes in CKD patients.

Over the past half a century, researchers have focused on the relationship between conventional cardiovascular risk factors and cardiovascular events in CKD patients. However, CKD patients present with unique cardiovascular challenges, and thus the need to explore non-traditional risk factors in this patient population has emerged. It has recently been reported that hyperphosphatemia, such as that occurring in CKD-mineral bone disease (CKD-MBD), may be a risk factor for the accelerated onset of cardiovascular disease in CKD patients (2–7).

A recent double-blind crossover interventional trial found that the acute elevation of serum phosphorus provoked endothelial dysfunction in CKD patients (8). In addition, a clinical trial found increased post-prandial serum phosphorus and significantly decreased flow-mediated dilation in 11 healthy men with a high-phosphorous diet. In an *in vitro* experiment, high phosphorus levels inhibited nitric oxide (NO) production, resulting in increased reactive oxygen species release and endothelial NO synthase inactivation via conventional protein kinase C, ultimately leading to impaired vasodilation. Based on these results, most advanced CKD patients are prescribed oral phosphate binders to lower their serum level of phosphorus; nonetheless, cardiovascular mortality in CKD patients remains high (9).

Meanwhile, EndoPAT is a useful device to assess endothelial dysfunction in clinical research as the test is easy to perform, not operator-dependent, and with comprehensive automatic analysis. The EndoPAT measures plethysmographical changes of vascular pressure in the finger by detecting arterial pulsation, and translates the signal into peripheral arterial tone. Several studies have shown the efficacy of peripheral artery tonometry (PAT) for assessing the endothelial function of peripheral vessels (10, 11). Peripheral tissue perfusion using laser Doppler flowmetry (LDF) accompanied by iontophoresis of acetylcholine and sodium nitroprusside, a well-known technology for estimation of perfusion and use in functional testing of endothelium-dependent microvascular reactivity (12). LDF provides a continuous record of the microvascular blood flow and is therefore an excellent method to measure microvascular blood flow changes and microvascular perfusion. Microvascular reactivity is a sensitive marker for endothelial dysfunction in superficial vessels (13).

Therefore, we investigated whether a normal phosphorus level can cause endothelial dysfunction and how the major factors involved in mineral metabolism, including serum phosphorus, parathyroid hormone (PTH), and fibroblast growth factor 23 (FGF23) levels, affect endothelial dysfunction in CKD patients, using EndoPAT and iontophoresis with LDF.

## Methods

### Study Design

This was a single-center, cross-sectional study. From January 2018 to December 2020, 85 patients with non-dialysis CKD were recruited from the outpatient clinic at the Ewha Womans University Mokdong hospital in Seoul, Korea. To be included in the study, patients were required to be aged between 18 and 65 years, have CKD stage 1–4, and have a normal reference serum level of phosphorus (2.3–4.5 mg/dL) for at least 3 months prior to enrollment. Patients with New York Heart Association class III/IV heart failure, acute myocardial infarction, severe peripheral arterial disease, decompensation in the past 3 months, malignancy in the past 5 years, hepatic insufficiency, acute infectious disease in the last 30 days, or who were pregnant or breastfeeding were excluded. Enrollment information and past medical history data were obtained from patient medical records. To evaluate endothelial function, iontophoresis with laser doppler flowmetry (ILDF) and EndoPAT-2000 (Peripheral Artery Tonometer; Itamar Medical, Israel) analysis were performed after the patients had fasted for at least 8 h. Each subject was instructed to avoid food, drugs, tobacco, alcohol, coffee, and tea 10 h prior to the test. The study protocol was approved by the hospital's ethics committee (IRB no. ECT12–02A-28) and written informed consent was obtained from all subjects prior to participation in the study. All clinical investigations were conducted in accordance with the 2013 Declaration of Helsinki.

### Iontophoresis With Laser-Doppler Flowmetry (ILDF)

Laser-doppler flowmetry is a non-invasive method used to determine endothelial dysfunction according to skin microvasculature function (14), and it is based on diffusion and refraction of laser beam light (15). LDF measures cutaneous blood perfusion using the Doppler shift principle, which is caused by moving red blood cells scattering light (16). Iontophoresis employs electrically repulsive forces to deliver a locally applied drug through the skin for therapeutic and diagnostic purposes (17). When coupled with LDF, iontophoresis enables the detection of changes in skin blood flow in response to vasoactive drug administration. The materials and techniques used for ILDF in the present study are in accordance with previous research (18). Briefly, during an acclimating period, patients sat in a comfortable chair for 20 min in a temperature-controlled room (22–24°C). Afterwards, an alcohol-soaked cotton pad was used to clean the skin of the forearm, and two drug delivery chamber electrodes (PF 383; Perimed, Järfälla, Sweden) were attached for delivering acetylcholine (Ach) and sodium nitroprusside (SNP) onto the skin. The drug-delivery electrodes (PF 384; Perimed) were attached 10 mm from the volar aspect of each drug delivery chamber to provide the current needed for Ach and SNP delivery. The two paired drug delivery chambers and drug delivery electrodes were located to the forearm at a distance of 10 cm. Then, 0.05 mL each of 1% solutions of Ach and SNP were injected into the anodal and cathodal chambers to measure endothelial-dependent and endothelial-independent responses, respectively (19). Next, the laser-Doppler probe (PF 408; Perimed) was connected to the LDF (PF 4001; Perimed) and fixed within the drug chamber to explore the same small area of skin. Laser-Doppler output was recorded continuously using arbitrary perfusion units (PU). After recording 5 min of stable baseline perfusion, Ach and SNP dose-response curves were obtained with stepwise current applications (20). Ach was delivered in six doses at 0.1 mA for 20 s each, followed by another two doses at 0.2 mA for 20 s each with a 180 s interval between the two successive doses. The absolute maximal response was defined as the flow rate reached after the last drug delivery. To eliminate baseline variability, the blood flow responses to locally delivered Ach and SNP were expressed as ratios of response PU to baseline PU; if the ratio was <100, it was classified as endothelial dysfunction.

### Measuring Peripheral Microvascular Function Using the EndoPAT-2000

The EndoPAT-2000 is a non-operator-dependent, non-invasive device used to assess microvascular function. It records post-ischemic reactive hyperemia, known as the peripheral arterial tone signal, using plethysmographic probes placed on the index finger of each hand. Prior to taking measurements, the patients were instructed to rest in a supine position for a minimum of 20 min in the same room where ILDF was performed. Each recording consisted of 5 min of baseline measurements, 5 min of occlusion measurements, and 5 min of post-occlusion measurements (hyperemic period). The occlusion pressure was at least 60 mmHg above the patient's systolic blood pressure (200–230 mmHg). Occlusion of the brachial artery was performed on the non-dominant upper arm. After 5 min of inflation the cuff was deflated, which induced reactive hyperemia. During this hyperemic period, flow measurements were recorded and the response to reactive hyperemia was calculated automatically by the software system. A reactive hyperemia index (RHI) was created using the post- and pre-occlusion values and normalized to measurements from the contralateral arm, which served as a control for non-endothelial-dependent systemic effects (10). An RHI value <1.67 was used as a cut-off value to diagnose endothelial dysfunction (11).

### Biochemical Analysis

Venous blood samples were collected from each patient prior to measuring endothelial function after overnight fasting (at least 8 h). Standard laboratory tests included complete blood cell counts and routine blood chemistry, including serum albumin, creatinine, triglycerides, total cholesterol, low density lipoprotein (LDL) cholesterol, and uric acid, activated vitamin D were collected using automated techniques. Values of estimated GFR (eGFR) were calculated with a new equation proposed by investigators at the Chronic Kidney Disease Epidemiology (CKD-EPI) Collaboration. Baseline phosphate levels were measured with an ammonium molybdate assay on the Olympus AU1000 auto-analyzer (normal range: 2.5–4.5 mg/dL). Intact PTH (iPTH) was measured using a radioimmunometric assay (normal range: 15–65 pg/mL; Scantibodies, Santee, CA, USA). Serum VCAM-1 levels were quantified using an ELISA kit designed to quantitatively measure the human soluble VCAM-1 concentration in serum (Human sVCAM-1 Immunoassay; R&D systems, Minneapolis, MN). Plasma FGF23 concentrations were measured using an ELISA assay (concentration detection range: 3–800 pg/mL) with a commercial reagent (Kainos Laboratories, Tokyo, Japan). The serum VCAM-1 and plasma FGF23 concentrations were measured twice and then averaged.

### Statistical Analysis

Because FGF23, VCAM-1, and the values from vascular measurements were not normally distributed, these variables were logarithmically transformed before further analysis. Variables correlated with endothelial dysfunction were evaluated by a univariate linear regression analysis and variables found to be significant, including eGFR, sex, albumin, and hemoglobin, were further analyzed with a multivariate stepwise linear regression analysis. The SPSS statistical package for Windows was used for all analyses (ver. 20.0; SPSS Inc., Chicago, IL, USA). A *P-*value of < 0.05 was considered statistically significant.

Mediation analyses were performed, when appropriate, based on the multivariate regression results to assess the hypothesized associations between PTH and endothelial dysfunction. Specifically, the mediation analysis was performed when the exposure was significantly correlated to the mediator or endothelial dysfunction, or when mediator was significantly related to endothelial dysfunction. Indirect effects and confidence intervals were estimated by bootstrapping with 5,000 resamples using the PROCESS Statistical Package for SPSS (PROCESS ver. 2 and SPSS version 27.0; SPSS Inc.). Statistically significant mediation was considered established when the indirect effect was significantly different from zero.

## Results

### Baseline Characteristics

Characteristics of the CKD patients are listed in Table 1. Of the 85 patients included in the study, 57.6% (*n* = 49) were males. The mean age was 49.5 years and the mean body mass index was 24.5 kg/m^2^. The etiology of CKD in this study included hypertension (18.8%), diabetes mellitus (12.9%), and glomerulonephritis (40.0%). Hypertension was the most common comorbidity during the CKD management follow-up period (56.5%), followed by dyslipidemia (32.9%), and diabetes mellitus (17.5%). Most patients were taking either angiotensin-converting enzyme inhibitors (15.3%) or angiotensin receptor blockers (63.5%) without concurrent use of angiotensin blockade. Approximately half of the patients were taking a statin (44.7%) and a calcium carbonate was prescribed for three patients for calcium supplement.

**Table 1:** Baseline characteristics.

**Variables**	**Number (%)**
Age, years, mean (range)	49.5 (24-65)
Male, *n* (%)	49 (57.6)
BMI (kg/m^2^)	24.5 ± 4.1
**Cause of CKD**, ***n*** **(%)**	
Hypertension	16 (18.8)
Diabetes mellitus	11 (12.9)
Glomerulonephritis	34 (40.0)
Others	24 (28.2)
**Comobidities**, ***n*** **(%)**	
Hypertension	48 (56.5)
Diabetes mellitus	15 (17.6)
Dyslipidemia	28 (32.9)
Cardiovascular disease	5 (5.9)
Cerebrovascular disease	3 (3.5)
**Current medication**, ***n*** **(%)**	
Beta blocker	12 (14.1)
Angiotensin-converting enzyme inhibitor	13 (15.3)
Angiotensin receptor blocker	54 (63.5)
Statin	38 (44.7)
Phosphorus binder	3 (3.5)

*Total number of subjects were 85 and data were presented as number (%) or mean ± standard deviation. BMI, body mass index*.

### Biochemical and Vascular Assessments

The biochemical and vascular assessments are presented in Table 2. The average serum phosphorus concentration was 3.5 ± 0.4 mg/dL, range 2.5 −4.5 mg/dL and corresponding mean eGFR was 62.5 mL/min/1.73 m^2^, ranging from 15.6 to 120.9 mL/min/1.73 m^2^. The average of intact PTH, log transformed FGF 23, and log transformed VCAM-1 were 62.1 ± 40.8 pg/mL, 4.7 ± 1.7, 5.9 ± 0.3, respectively.

**Table 2:** Biochemical and vascular assessment.

**Variables**	Mean ±standard deviation (*n*)*
**Laboratory finding**	
Hemoglobin (g/dL)	13.5 ± 1.8 (83)
Estimated glomerular filtration rate (mL/min/1.73 m^2^)	62.5 ± 24.0 (85)
Albumin (g/dL)	4.0 ± 0.5 (81)
Uric acid (mg/dL)	6.1 ± 1.8 (57)
Triglycerides (mg/dL)	138.9 ± 75.5 (84)
Total cholesterol (mg/dL)	173.0 ± 36.8 (84)
Low density cholesterol (mg/dL)	97.2 ± 27.0 (81)
Phosphorus (mg/dL)	3.5 ± 0.4 (85)
Total calcium (mg/dL)	8.8 ± 0.4 (85)
Activated vitamin D, pg/ml	32.2 ± 17.5 (19)
Intact parathyroid hormone (pg/mL)	62.1 ± 40.8 (37)
Log FGF23 (pg/mL)	4.7 ± 1.7 (60)
Log VCAM-1 (ng/mL)	5.9 ± 0.3 (60)
**Vascular assessment**	
SBP, mmHg	122.8 ± 14.4
DBP, mmHg	74.2 ± 9.9
Log Acetylcholine-induced iontophoresis (ratio of response to baseline)	13.2 ± 9.1
Log Nitropurusside-induced iontophoresis (ratio of response to baseline)	11.5 ± 6.2
Reactive hyperemia index (%) measured by endoPAT	2.2 ± 0.6

^*^Number of cases.

*Data were presented as mean ± standard deviation or number (%)*.

*FGF23, fibroblast growth factor 23; SBP, systolic blood pressure; DBP, diastolic blood pressure*.

On the day of vascular assessment, the average systolic blood pressure and diastolic pressure were 122.8 ± 14.4 and 74.2 ± 9.9 mmHg, respectively. For the endothelial function test, endothelial dysfunction was observed in all subjects as the ratio of their response PU to baseline PU value measured according to Ach-induced ILDF <100. The log-transformed responses to Ach- and SNP-induced iontophoresis and RHI values were 13.2 ± 9.1, 11.5 ± 6.2, and 2.2 ± 0.6, respectively. Endothelial dysfunction, defined as an RHI value <1.67, was detected in 23 patients (27%).

**Figure 1:**
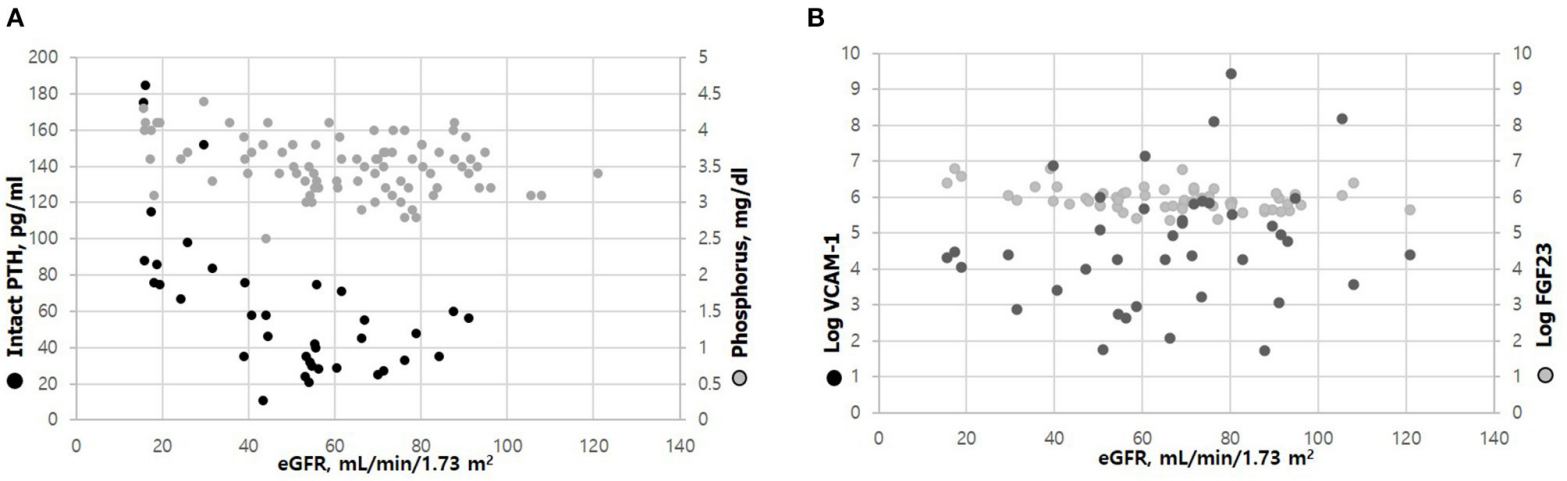
Scatter plot of the relationships between eGFR and iPTH; phosphorus **(A)**, and Log FGF23; Log VCAM-1 **(B)**. Phosphorus, iPTH, and Log VACM-1 were correlated with eGFR significantly (*r* = −0.32. *p* = 0.003 for phosphorus; *r* = −0.631, *p* < 0.001 for PTH; *r* = −0.405, *p* < 0.001 for Log VACM-1. There was no significant trend of Log FGF 23 in relation to eGFR **(B)**.

A scatter plot of the relationship between eGFR and PTH, serum phosphorus, log FGF 23, and log VCAM-1 is shown in Figure 1. As eGFR decreased, phosphorus (*r* = −0.32, *P* = 0.003) and iPTH (*r* = −0.631, *P* < 0.001) significantly increased, although serum phosphorus levels were within normal limits (Figure 1A). Log VCAM-1 was negatively correlated with eGFR (*r* = −0.405, *P* < 0.001), while the change in FGF 23 was not significant.

### Associations Between Phosphorus, iPTH, FGF23, VCAM-1 and Endothelial Function Assessments (Table 3)

In the crude model, eGFR (r = 0.22, *P* = 0.043), iPTH (*r* = −0.46, *P* = 0.005), and log-transformed VCAM-1 (*r* = −0.36, *P* = 0.005) were correlated with log-transformed Ach-induced iontophoresis. NSP-induced iontophoresis was not significantly correlated with serum phosphorus, iPTH, FGF23, or VCAM-1, however, serum phosphorus (*r* = 0.32, *P* = 0.003) and iPTH (*r* = 0.39, *P* = 0.016) were correlated with RHI.

**Table 3:** Crude association between phosphorus, iPTH, FGF23, VCAM-1 and endothelial function assessments by Pearson correlation.

**Variables**	**Log Acetylcholine-induced iontophoresis**		**Log Nitropurusside-induced iontophoresis**		**Reactive hyperemia**	
	**(ratio of response to baseline)**		**(ratio of response to baseline)**		**index (%)**	
	***r***	***P*-value**	***r***	***P*-value**	***r***	***P*-value**
eGFR	0.22	0.04	0.03	0.76	−0.17	0.11
Phosphorus	−0.06	0.60	0.02	0.83	0.32	0.003
PTH	−0.46	0.005	−0.21	0.20	0.39	0.02
Log FGF23	−0.15	0.37	−0.05	0.76	0.03	0.86
Log VCAM-1	−0.36	0.005	−0.18	0.18	−0.02	0.91

*eGFR, Estimated glomerular filtration rate*.

### Associations Between iPTH, Serum Phosphorus, and RHI (Table 4)

When adjusted for eGFR, iPTH (β = 0.006, SE = 0.003, *P* = 0.048) and serum phosphorus (β = 0.464, SE = 0.173, *P* = 0.009) were significant predictors for RHI. Potential cofounders such as age, hypertension, diabetes, and covariants including, sex, serum albumin level, and hemoglobin, significant in the univariate analysis (Supplementary Table 1), were adjusted in model 2. iPTH remained statistically significant after the multiple regression analysis (β = 0.008, SE = 0.003, *P* = 0.017).

**Table 4:** Associations of PTH and phosphorus with endoPAT by multiple linear regression analyses.

	**Model 1**			**Model 2**		
	**β**	**SE**	***p*-value**	**β**	**SE**	***p*-value**
iPTH	0.006	0.003	0.048	0.008	0.003	0.017
phosphorus	0.464	0.173	0.009	0.143	0.314	0.660

*Model 1: Adjusted for eGFR*.

*Model 2: Adjusted for eGFR, age, history of hypertension and diabetes, sex, albumin, and hemoglobin*.

*eGFR, Estimated glomerular filtration rate; SE, standard error*.

### Associations Between iPTH, Serum Phosphorus, and Ach-Induced Iontophoresis (Table 5)

Similarly, when adjusted by eGFR, iPTH (β = −0.006, SE = 0.003, *P* = 0.026) and VCAM-1 (β = −0.581, SE = 0.226, *P* = 0.013) remained significant predictors for Ach-induced iontophoresis in model 1. After adjusting for potential cofounders (age, diabetes) and covariants, including eGFR, history of hypertension and dyslipidemia, use of beta blockers, and hemoglobulin level, that were significant in the univariate analyses (Supplementary Table 1), VCAM-1 remained statistically significant in model 2 (β = −0.218, SE = 0.092, *P* = 0.039).

**Table 5:** Associations of PTH and VCAM-1 with Acetylcholine-induced iontophoresis^*^ (ratio of response to baseline) by multiple linear regression analyses.

	**Model 1**			**Model 2**		
	**β**	**SE**	***p*-value**	**β**	**SE**	***p*-value**
PTH	−0.006	0.003	0.026	−0.011	0.005	0.078
VCAM-1*	−0.581	0.226	0.013	−0.218	0.092	0.039

^*^Log-transformed values were used for analyses.

*Model 1: Adjusted for eGFR*.

*Model 2: Adjusted for eGFR, age, history of hypertension, diabetes and dyslipidemia, use of beta blocker, and hemoglobin*.

*eGFR, Estimated glomerular filtration rate; SE, standard error; VCAM-1, vascular cell adhesion molecule-1*.

### Mediation Analysis

**Figure 2:**
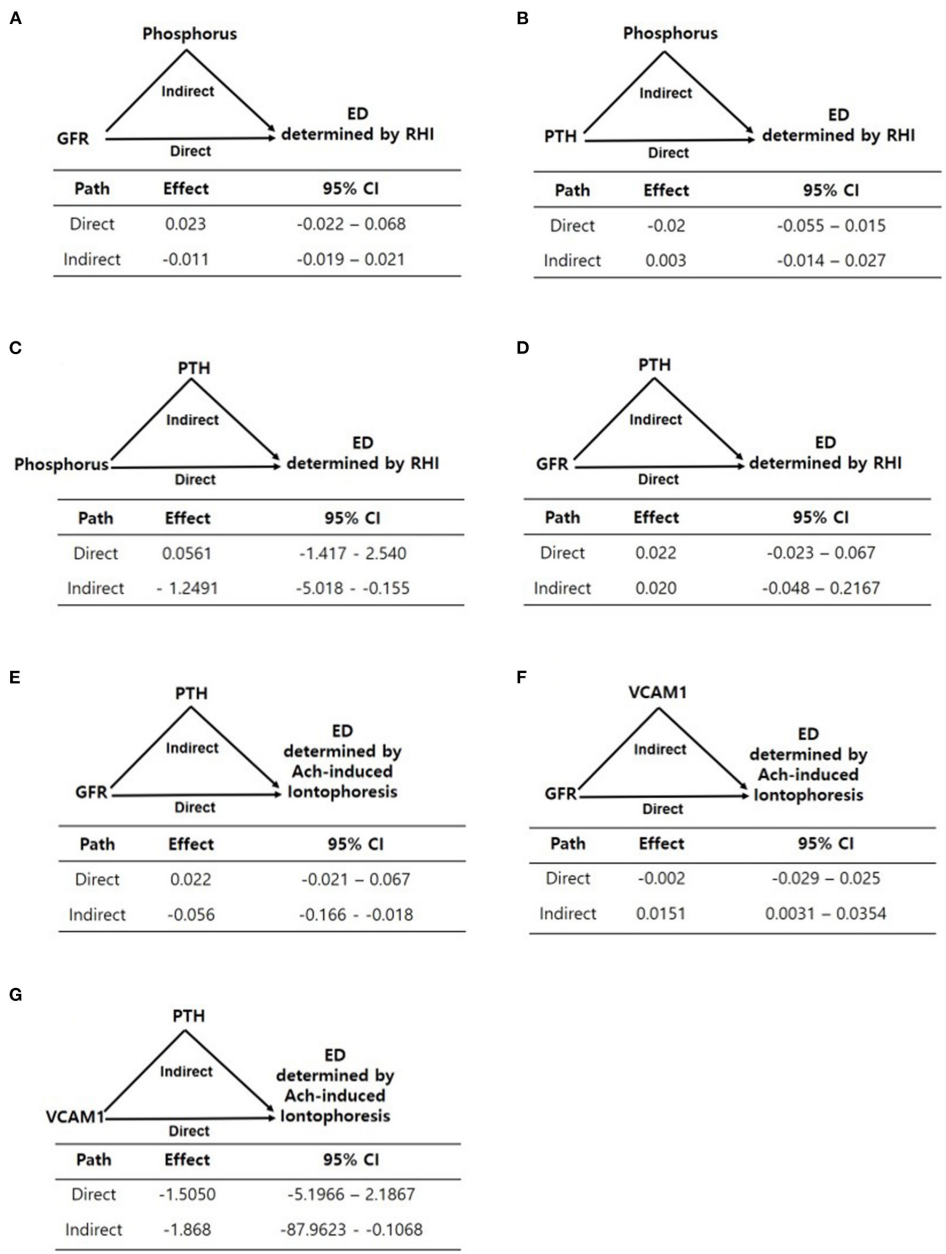
**(A–G)** Mediation analyses of the associations between variables including eGFR, phosphorus, PTH, and endothelial dysfunction determined by EndoPAT and laser doppler iontophoresis. Path models and mediation analyses describe mediation of the association between variables and endothelial dysfunction through endothelial-related biomarkers individually. Path effects are reported as Odds-ratio scale of natural log-transformed values of biomarkers.

The results of the mediation analysis are summarized in Figure 2. We divided the patients into two groups according to mean log-transformed Ach-induced iontophoresis (ratio of response to baseline) and the RHI cutoff value and defined the lower of the two as endothelial dysfunction. Because FGF23 and serum phosphorus were not associated with RHI and Ach-induced iontophoresis values, respectively, we did not test whether FGF23 and serum phosphorus showed any mediation in each endothelial dysfunction assessment. Instead, we tested whether serum phosphorus, iPTH, and eGFR had mediation or residual direct effects on endothelial function assessed by RHI and ILDF. Because phosphorus elevation was fundamentally linked with a decrease in eGFR, eGFR was included as an independent variable in the mediation analysis. In addition, phosphorus and iPTH, which remained significant in the multivariate analysis (Tables 4, 5), were replaced alternatively as independent and mediator variables in each model to yield direct and mediational effects. As a result, the direct effect of eGFR was not significant for endothelial dysfunction measured using RHI and ILDF (Figures 2A,E). There were no direct effects for serum phosphorus and iPTH on endothelial dysfunction measured using RHI (Figures 2B,C), however, there was a significant mediation effect for iPTH (Figure 2C).

For endothelial dysfunction measured using Ach-induced iontophoresis, eGFR's indirect effect mediated by iPTH and VCAM-1 was significant, as opposed to eGFR's direct effect (Figures 2E,F), and iPTH significantly mediated the effect of VCAM-1 on endothelial dysfunction (Figure 2G).

## Discussion

In the present study, we showed that eGFR is inversely correlated with both serum phosphorus levels and PTH, although serum phosphorus levels were within normal limits (Figure 1). It is well-established that as eGFR declines, serum phosphorus is retained and PTH rises. Even in the early stages of CKD, phosphorus retention stimulates FGF23 and PTH secretion, which in turn suppresses renal phosphate reabsorption and magnifies renal phosphate excretion. Notably, there was a positive correlation between PTH and phosphorus levels when PTH was elevated and phosphorus was within normal limits. Limited information on overall mineral metabolism, such as whether PTH was well-maintained prior to recruitment, might have had a confounding effect on our interpretation of the relationship between PTH and phosphorus. However, we found that both iPTH and phosphorus were associated with endothelial dysfunction despite phosphorus being within a normal reference range. Similarly, a previous study found that newly diagnosed hypertensive patients with normal renal function showed a vasodilating response to Ach in the forearm that was inversely and independently related to serum phosphorus levels, even when serum phosphorous was within normal limits. In that study, a group with phosphorus levels 3.9–4.2 mg/dL had a 1.7-fold greater chance of having endothelial dysfunction than those with levels of 2.7–3.9 mg/dL (21). In an observational study of healthy Korean patients with normal renal function, a lower concentration of serum phosphorus (within the normal range) was associated with reduced calcification of the coronary artery (22). Taken together, these results suggest that even a relatively high serum level of phosphorus within the normal range could be a risk factor for cardiovascular disease.

In the current study, we observed that as eGFR decreased, increased iPTH and phosphorus were related to endothelial dysfunction (Table 3). Both iPTH and phosphorus were associated with endothelial dysfunction measured using RHI, and the relationships remained even after an adjustment for covariates, including eGFR (Table 4). iPTH was significantly related to Ach-induced ILDF even after eGFR was adjusted (Table 5). However, when adjusted for eGFR and the other significant covariates in each model, the independent association between iPTH and Ach-induced ILDF disappeared, whereas the association between iPTH and RHI remained. This discrepancy may be due to the varied contribution of iPTH to each measurement of endothelial dysfunction, or the interplay between iPTH and CKD-MBD-related biomarkers.

Next, we investigated how iPTH mediates other CKD-MBD biomarkers to assess endothelial dysfunction. Although both phosphorus and iPTH were associated with endothelial dysfunction in the eGFR-adjusted model, the strength of the association between both phosphorus and iPTH with endothelial dysfunction was reduced in the multiple factors-adjusted model. However, the mediation analysis showed that iPTH mediated the associations between eGFR, phosphorus, and endothelial dysfunction. The association between VCAM-1 and endothelial dysfunction was mediated by iPTH as well. Taken together, iPTH served as a major regulator in endothelial dysfunction, either directly or indirectly, which suggests that iPTH mediates the association between CKD-MBD biomarkers and endothelial dysfunction.

Meanwhile, FGF23 is a phosphaturic hormone produced by osteocytes and osteoblasts in response to hyperphosphatemia. Recent studies indicate that increased FGF23 levels are associated with hindered NO bioavailability in the endothelium and impaired endothelium-dependent vasodilation (23, 24). Unfortunately, the effect of FGF23 on endothelial dysfunction was not verified in the present study, probably because eGFR was not correlated with FGF23. In a previous study, normophospahtemic CKD patients treated with calcium acetate and sevelamer hydrochloride in both arms showed decreased serum levels of FGF23 over a treatment period of 6 weeks (25). Because these findings were not attributed to the serum level of phosphorus, serum calcium was suggested to be another determinant for FGF23 based upon its correlation with FGF23 independent of phosphorus in primary hyperparathyroidism (26). In our study, calcium and phosphorus levels were not correlated, and data on these and the corresponding FGF23 levels prior to enrollment were limited. Thus, further studies on the factors controlling FGF23 production are warranted.

Regarding methodologic considerations, iontophoresis with Ach and SNP combined with laser doppler perfusion measurements have been used to assess cutaneous microcirculation and have been validated in several clinical studies. Previous studies have demonstrated that the response to Ach-dependent iontophoresis is impaired in patients with type 1 diabetes (27) and in patients with an increased risk for coronary heart disease (28, 29). Ach-induced vasodilation depends on the presence of intact vascular endothelium, whereas SNP acts directly on vascular smooth muscle cells, which are known as endothelium-dependent and endothelium-independent vasodilation, respectively. In particular, Ach-induced vasodilation is mediated by the generation of NO, prostaglandins, and endothelial-derived hyperpolarizing factors. Thus, endothelial dysfunction can be evaluated by comparing Ach- and SNP-induced vasodilation. Ach and SNP can be administered transdermally by iontophoresis and increases in perfusion can be quantified using LDF.

In agreement with previous studies in which CKD and PTH implicated impaired NO-mediated vasodilation, our results indicate that eGFR and elevated iPTH and VCAM-1 are related to a decrease in Ach-induced vasodilation (Table 3). Endothelium-derived relaxing factors, mainly NO production, are suppressed in patients and animals with CKD (30–33). Indeed, the renal synthesis of the NO precursor L-arginine is reduced in CKD patients (34). Furthermore, the transport of L-arginine into endothelial cells and the shunting of this amino acid into other pathways, such as those involving arginase, contribute to the reduced availability of this NO precursor. Regarding iPTH, *in vivo* study showed chronic renal insufficiency is associated with reduced endothelial nitric oxide synthase expression and reduced NO generation, an effect that is reversed by parathyroidectomy (35). Another *in vitro* study investigated the effect of high levels of iPTH on the proliferation of endothelial cells using the serum of 15 patients with secondary hyperparathyroidism (SHPT), 10 patients with CKD 5 without SHPT, and 15 healthy volunteers (36). The study found that the proliferation of human umbilical vein endothelial cells was inhibited by the serum obtained from SHPT patients and CKD 5 patients without SHPT, and the inhibitory effects of the SHPT serum were the most marked when the synthesis of NO was significantly decreased. In the present study, we confirmed that PTH induced endothelial-dependent endothelial dysfunction using ILDF. Further studies will be needed to clarify whether NO, prostaglandins, or endothelial-derived hyperpolarizing factors are involved in the mechanism by which PTH induces endothelial dysfunction.

VCAM-1 is considered an endothelial-specific biomarker and an important adhesion molecule that is upregulated during endothelial activation (37). Because decreased NO activity and increased endothelial expression of the VCAM-1 gene are functionally interrelated (38), we expected that VCAM-1 would be related to decreased Ach-induced vasodilation. In terms of SNP-induced vasodilation, any factors involved in mineral metabolism were not associated with SNP-induced vasodilation, which is consistent with previous studies (14, 15, 39).

The digital vascular response to reactive hyperemia is predominantly dependent on NO-mediated vasodilation in healthy subjects (40) and is attenuated in patients with coronary microvascular endothelial dysfunction (11). In the present study, as expected, serum phosphorus and iPTH were correlated with RHI in our crude model. After adjusting for multiple significant factors, only iPTH remained significantly associated with RHI.

Taken together, our data suggest that ILDF and EndoPAT-2000 measurements appear to analyze different aspects of vascular function in selected vascular beds and vessel sizes. Thus, we suggest cautiously that biomarkers of mineral metabolism, such as serum phosphorus and iPTH, involve impaired vasodilation based on different vascular beds and vessel sizes. Finally, our data confirm that endothelial dysfunction is a multifactorial and complex disorder that occurs in CKD patients with an arbitrarily set normal range of serum phosphorus levels.

Our study had some limitations that should be considered. Given its small patient population and cross-sectional design, the causal relationships in the present study cannot be determined. In addition, the assessments were only performed once and were not based on a time series. Furthermore, regarding the calcium-PTH-vitamin D axis, vitamin D deficiency is a pivotal change in CKD-MBD, in which vitamin D receptors are expressed in the vascular endothelium. Vitamin D deficiency in CKD patients has been suggested to be independently associated with impaired FMD (41) and vitamin D supplementation improves endothelial dysfunction in such patients (42, 43). In the present study, the lack of data on vitamin D levels may confound our observed effects on endothelial dysfunction. Another limitation is that there was no objective reference for defining endothelial dysfunction assessed by ILDF, particularly in CKD patients. We used an arbitrary ratio of responses to baseline perfusion units to describe blood volume changes (18, 44). Because ILDF is non-invasive and has high reproducibility with an intra-assay variation coefficient of <6% (45), it is considered appropriate for evaluating microvascular function. Finally, to isolate and clarify the effect of serum phosphorus levels and iPTH on endothelial function, while currently available statistical methods were used, our results are insufficient to fully explain the complex effects of CKD-MBD biomarkers on endothelial dysfunction in CKD patients. Despite these limitations, this is the first study to investigate the effects of CKD-MBD biomarkers on microvascular function in CKD patients with normal serum phosphorus levels.

In conclusion, a normal reference range of serum phosphorus levels and corresponding elevated PTH values is associated with endothelial dysfunction in non-dialysis CKD patients. Further studies are needed to determine whether adjusting phosphorus levels is necessary in CKD patients.

## Data Availability Statement

The original contributions presented in the study are included in the article/Supplementary Material, further inquiries can be directed to the corresponding author/s.

## Ethics Statement

The studies involving human participants were reviewed and approved by the Institutional Review Board of Ewha. The patients/participants provided their written informed consent to participate in this study.

## Author Contributions

SL contributed to the conception or design of the work and drafted the manuscript. S-JK and SL contributed to the acquisition, analysis, or interpretation of data for the work. S-JK critically revised the manuscript. All gave final approval and agree to be accountable for all aspects of work ensuring integrity and accuracy.

## Conflict of Interest

The authors declare that the research was conducted in the absence of any commercial or financial relationships that could be construed as a potential conflict of interest.

## Publisher's Note

All claims expressed in this article are solely those of the authors and do not necessarily represent those of their affiliated organizations, or those of the publisher, the editors and the reviewers. Any product that may be evaluated in this article, or claim that may be made by its manufacturer, is not guaranteed or endorsed by the publisher.

## References

[1] Chapter 3: Morbidity and mortality in patients with Ckd. Am J Kid Dis. (2017) 69:S67–92. 10.1053/j.ajkd.2017.01.012

[2] Betriu A Martinez-Alonso M Arcidiacono MV Cannata-Andia J Pascual J Valdivielso JM. Prevalence of subclinical atheromatosis and associated risk factors in chronic kidney disease: the nefrona study. Nephrol Dia Transplant. (2014) 29:1415–22. 10.1093/ndt/gfu038 24586070

[3] Ganesh SK Stack AG Levin NW Hulbert-Shearon T Port FK. Association of elevated serum Po4, Ca × Po4 product, and parathyroid hormone with cardiac mortality risk in chronic hemodialysis patients. J Am Soc Nephrol. (2001) 12:2131–8. 10.1681/ASN.V12102131 11562412

[4] Achinger SG Ayus JC. Left ventricular hypertrophy: is hyperphosphatemia among dialysis patients a risk factor? J Am Soc Nephrol. (2006) 17(12 Suppl 3):S255–61 10.1681/ASN.2006080923 17130271

[5] Block GA Hulbert-Shearon TE Levin NW Port FK. Association of serum phosphorus and calcium X phosphate product with mortality risk in chronic hemodialysis patients: a national study. Am J Kidney Dis. (1998) 31:607–17. 10.1053/ajkd.1998.v31.pm9531176 9531176

[6] Block GA Klassen PS Lazarus JM Ofsthun N Lowrie EG Chertow GM. Mineral metabolism, mortality, and morbidity in maintenance hemodialysis. J Am Soc Nephrol. (2004) 15:2208–18. 10.1097/01.ASN.0000133041.27682.A2 15284307

[7] Kestenbaum B Sampson JN Rudser KD Patterson DJ Seliger SL Young B. Serum phosphate levels and mortality risk among people with chronic kidney disease. J Am Soc Nephrol. (2005) 16:520–8. 10.1681/ASN.2004070602 15615819

[8] Shuto E Taketani Y Tanaka R Harada N Isshiki M Sato M. Dietary phosphorus acutely impairs endothelial function. J Am Soc Nephrol. (2009) 20:1504–12. 10.1681/ASN.2008101106 19406976 PMC2709683

[9] Saran R Robinson B Abbott KC Agodoa LYC Bragg-Gresham J Balkrishnan R. Us Renal data system 2018 annual data report: epidemiology of kidney disease in the United States. Am J Kidney Dis. (2019) 73(3 Suppl 1):A7–8. 10.1053/j.ajkd.2019.01.001 30798791 PMC6620109

[10] Axtell AL Gomari FA Cooke JP. Assessing endothelial vasodilator function with the endo-pat. J Vis Exp. (2010) 44:2167. 10.3791/2167 20972417 PMC3143035

[11] Bonetti PO Pumper GM Higano ST Holmes DR Jr Kuvin JT Lerman A. Noninvasive identification of patients with early coronary atherosclerosis by assessment of digital reactive hyperemia. J Am Coll Cardiol. (2004) 44:2137–41. 10.1016/j.jacc.2004.08.062 15582310

[12] Turner J Belch JJ Khan F. Current concepts in assessment of microvascular endothelial function using laser doppler imaging and iontophoresis. Trends Cardiovasc Med. (2008) 18:109–16. 10.1016/j.tcm.2008.02.001 18555183

[13] Houben A Martens RJH Stehouwer CDA. Assessing microvascular function in humans from a chronic disease perspective. J Am Soc Nephrol. (2017) 28:3461–72. 10.1681/ASN.2017020157 28904002 PMC5698072

[14] Cupisti A Rossi M Placidi S Caprioli R Morelli E Vagheggini G. Responses of the skin microcirculation to acetylcholine and to sodium nitroprusside in chronic uremic patients. Int J Clin Lab Res. (2000) 30:157–62. 10.1007/s005990070015 11196074

[15] Babos L Járai Z Nemcsik J. Evaluation of microvascular reactivity with laser doppler flowmetry in chronic kidney disease. World J Nephrol. (2013) 2:77–83. 10.5527/wjn.v2.i3.77 24255889 PMC3832914

[16] Niwayama J Sanaka T. Development of a New method for monitoring blood purification: the blood flow analysis of the head and foot by laser doppler blood flowmeter during hemodialysis. Hemodial Int. (2005) 9:56–62. 10.1111/j.1492-7535.2005.01118.x 16191054

[17] Kalia YN Naik A Garrison J Guy RH. Iontophoretic drug delivery. Adv Drug Deliv Rev. (2004) 56:619–58. 10.1016/j.addr.2003.10.026 15019750

[18] Lee S Ryu JH Kim SJ Ryu DR Kang DH Choi KB. The relationship between magnesium and endothelial function in end-stage renal disease patients on hemodialysis. Yonsei Med J. (2016) 57:1446–53. 10.3349/ymj.2016.57.6.1446 27593873 PMC5011277

[19] Morris SJ Shore AC Tooke JE. Responses of the skin microcirculation to acetylcholine and sodium nitroprusside in patients with Niddm. Diabetologia. (1995) 38:1337–44. 10.1007/BF00401767 8582544

[20] Davis KR Ponnampalam J Hayman R Baker PN Arulkumaran S Donnelly R. Microvascular vasodilator response to acetylcholine is increased in women with pre-eclampsia. Bjog. (2001) 108:610–4. 10.1111/j.1471-0528.2001.00144.x 11426896

[21] Perticone M Maio R Sciacqua A Cimellaro A Andreucci M Tripepi G. Serum phosphorus levels are associated with endothelial dysfunction in hypertensive patients. Nutr Metab Cardiovasc Dis. (2016) 26:683–8. 10.1016/j.numecd.2016.02.003 27105871

[22] Park KS Chang JW Kim TY Kim HW Lee EK Kim HS. Lower concentrations of serum phosphorus within the normal range could be associated with less calcification of the coronary artery in koreans with normal renal function. Am J Clin Nutr. (2011) 94:1465–70. 10.3945/ajcn.110.001974 22030227

[23] Silswal N Touchberry CD Daniel DR McCarthy DL Zhang S Andresen J. Fgf23 directly impairs endothelium-dependent vasorelaxation by increasing superoxide levels and reducing nitric oxide bioavailability. Am J Physiol Endocrinol Metab. (2014) 307:E426–36. 10.1152/ajpendo.00264.2014 25053401 PMC4154070

[24] Yilmaz MI Sonmez A Saglam M Yaman H Kilic S Demirkaya E. Fgf-23 and vascular dysfunction in patients with stage 3 and 4 chronic kidney disease. Kidney Int. (2010) 78:679–85. 10.1038/ki.2010.194 20613714

[25] Oliveira RB Cancela AL Graciolli FG Dos Reis LM Draibe SA Cuppari L. Early control of Pth and Fgf23 in normophosphatemic Ckd patients: a new target in Ckd-Mbd therapy? Clin J Am Soc Nephrol. (2010) 5:286–91. 10.2215/CJN.05420709 19965540 PMC2827593

[26] Kobayashi K Imanishi Y Miyauchi A Onoda N Kawata T Tahara H. Regulation of plasma fibroblast growth factor 23 by calcium in primary hyperparathyroidism. Eur J Endocrinol. (2006) 154:93–9. 10.1530/eje.1.02053 16381997

[27] Khan F Elhadd TA Greene SA Belch JJ. Impaired skin microvascular function in children, adolescents, and young adults with type 1 diabetes. Diab Care. (2000) 23:215–20. 10.2337/diacare.23.2.215 10868834

[28] RG IJ de Jongh RT Beijk MA van Weissenbruch MM Delemarre-van de Waal HA Serné EH. Individuals at increased coronary heart disease risk are characterized by an impaired microvascular function in skin. Eur J Clin Invest. (2003) 33:536–42. 10.1046/j.1365-2362.2003.01179.x 12814388

[29] Khan F Patterson D Belch JJ Hirata K Lang CC. Relationship between peripheral and coronary function using laser doppler imaging and transthoracic echocardiography. Clin Sci (Lond). (2008) 115:295–300. 10.1042/CS20070431 18338981

[30] Wever R Boer P Hijmering M Stroes E Verhaar M Kastelein J. Nitric oxide production is reduced in patients with chronic renal failure. Arterioscler Thromb Vasc Biol. (1999) 19:1168–72. 10.1161/01.ATV.19.5.1168 10323766

[31] Aiello S Noris M Todeschini M Zappella S Foglieni C Benigni A. Renal and systemic nitric oxide synthesis in rats with renal mass reduction. Kidney Int. (1997) 52:171–81. 10.1038/ki.1997.317 9211360

[32] Erdely A Greenfeld Z Wagner L Baylis C. Sexual Dimorphism in the aging kidney: effects on injury and nitric oxide system. Kidney Int. (2003) 63:1021–6. 10.1046/j.1523-1755.2003.00830.x 12631083 PMC2793681

[33] Wagner L Riggleman A Erdely A Couser W Baylis C. Reduced nitric oxide synthase activity in rats with chronic renal disease due to glomerulonephritis. Kidney Int. (2002) 62:532–6. 10.1046/j.1523-1755.2002.00465.x 12110014 PMC2747491

[34] Baylis C. Arginine, arginine analogs and nitric oxide production in chronic kidney disease. Nat Clin Pract Nephrol. (2006) 2:209–20. 10.1038/ncpneph0143 16932427 PMC2756810

[35] Vaziri ND Ni Z Wang XQ Oveisi F Zhou XJ. Downregulation of nitric oxide synthase in chronic renal insufficiency: role of excess Pth. Am J Physiol. (1998) 274:F642–9. 10.1152/ajprenal.1998.274.4.F642 9575886

[36] Chen C Mao H Yu X Sun B Zeng M Zhao X. Effect of secondary hyperparathyroidism serum on endothelial cells and intervention with Klotho. Mol Med Rep. (2015) 12:1983–90. 10.3892/mmr.2015.3606 25873020

[37] Roumeliotis S Mallamaci F Zoccali C. Endothelial dysfunction in chronic kidney disease, from biology to clinical outcomes: a 2020 update. J Clin Med. (2020) 9:2359. 10.3390/jcm9082359 32718053 PMC7465707

[38] Khan BV Harrison DG Olbrych MT Alexander RW Medford RM. Nitric oxide regulates vascular cell adhesion molecule 1 gene expression and redox-sensitive transcriptional events in human vascular endothelial cells. Proc Natl Acad Sci USA. (1996) 93:9114–9. 10.1073/pnas.93.17.9114 8799163 PMC38604

[39] Iredahl F Löfberg A Sjöberg F Farnebo S Tesselaar E. Non-invasive measurement of skin microvascular response during pharmacological and physiological provocations. PLoS ONE. (2015) 10:e0133760. 10.1371/journal.pone.0133760 26270037 PMC4536230

[40] Nohria A Gerhard-Herman M Creager MA Hurley S Mitra D Ganz P. Role of nitric oxide in the regulation of digital pulse volume amplitude in humans. J Appl Physiol. (1985) (2006) 101:545–8. 10.1152/japplphysiol.01285.2005 16614356

[41] Dalan R Liew H Tan WKA Chew DE Leow MK-S. Vitamin D and the endothelium: basic, translational and clinical research updates. IJC Metabol Endocrine. (2014) 4:4–17. 10.1016/j.ijcme.2014.06.003

[42] Lundwall K Jörneskog G Jacobson SH Spaak J. Paricalcitol, microvascular and endothelial function in non-diabetic chronic kidney disease: a randomized trial. Am J Nephrol. (2015) 42:265–73. 10.1159/000441364 26496210

[43] Zoccali C Curatola G Panuccio V Tripepi R Pizzini P Versace M. Paricalcitol and endothelial function in chronic kidney disease trial. Hypertension. (2014) 64:1005–11. 10.1161/HYPERTENSIONAHA.114.03748 25259743

[44] Ryu JH Yu M Lee S Ryu DR Kim SJ Kang DH. Ast-120 improves microvascular endothelial dysfunction in end-stage renal disease patients receiving hemodialysis. Yonsei Med J. (2016) 57:942–9. 10.3349/ymj.2016.57.4.942 27189289 PMC4951472

[45] Tenland T Salerud EG Nilsson GE Oberg PA. Spatial and temporal variations in human skin blood flow. Int J Microcirc Clin Exp. (1983) 2:81–90. 6236158

