# Supplementary material for: Effects of Normal Reference Range of Phosphorus and Corresponding PTH on Endothelial Function in CKD Patients
Source: Front Med (Lausanne). 2022 Jul 12;9:935977. doi: 10.3389/fmed.2022.935977 (PMC9314747; doi:10.3389/fmed.2022.935977)
Supplement: Supplementary file 1 [file Table_1.DOCX]

Supplementary table 1. Univariate analysis of relationship between vascular assessment and covariant.

| Variables | Age | Male | Cause of CKD | HTN | CAD | CVD | DM | Dyslipidemia | SBP | DBP |
| --- | --- | --- | --- | --- | --- | --- | --- | --- | --- | --- |
| Correlation Coefficient | *r* (*p*-value) | *r* (*p*-value) | *r* (*p*-value) | *r* (*p*-value) | *r* (*p*-value) | *r* (*p*-value) | *r* (*p*-value) | *r* (*p*-value) | *r* (*p*-value) | *r* (*p*-value) |
| Reactive hyperemia index (%) | -0.01  (0.96) | 0.26  (0.02) | -0.06  (0.59) | 0.21  (0.70) | 0.08  (0.47) | 0.13  (0.25) | 0.04  (0.70) | 0.06  (0.56) | -0.07  (0.53) | -0.22  (0.50) |
| Log Acetylcholine-induced iontophoresis  (ratio of response to baseline) | -0.22  (0.40) | 0.17  (0.48) | 0.12  (0.27) | -0.29  (0.01) | -0.06  (0.57) | 0.10  (0.36) | -0.21  (0.06) | -0.27  (0.01) | -0.11  (0.29) | -0.05  (0.68) |
| Log Nitropurusside-induced iontophoresis (ratio of response to baseline) | -0.13  (0.23) | 0.36  (0.00) | -0.02  (0.86) | -0.12  (0.26) | -0.13  (0.22) | 0.04  (0.72) | -0.36  (0.00) | -0.02  (0.86) | 0.05  (0.65) | 0.16  (0.16) |

Supplementary table 1. *Continued*

| Variables | hemoglobin | eGFR | Triglyceride | Cholesterol | Albumin | Uric acid | LDL | Active vit D |
| --- | --- | --- | --- | --- | --- | --- | --- | --- |
| Correlation Coefficient | *r* (*p*-value) | *r* (*p*-value) | *r* (*p*-value) | *r* (*p*-value) | *r* (*p*-value) | *r* (*p*-value) | *r* (*p*-value) | *r* (*p*-value) |
| Reactive hyperemia index (%) | -0.25  (0.03) | -0.18  (0.10) | 0.24  (0.03) | 0.17  (0.12) | -0.21  (0.04) | 0.23  (0.08) | 0.07  (0.53) | -0.21  (0.39) |
| Log Acetylcholine-induced iontophoresis  (ratio of response to baseline) | 0.21  (0.04) | 0.20  (0.07) | -0.05  (0.68) | 0.05  (0.64) | 0.11  (0.31) | -0.13  (0.32) | 0.09  (0.43) | -0.30  (0.21) |
| Log Nitropurusside-induced iontophoresis (ratio of response to baseline) | 0.08  (0.49) | 0.04  (0.73) | -0.17  (0.11) | 0.20  (0.07) | 0.06  (0.58) | -0.04  (0.78) | 0.26  (0.02) | 0.33  (0.17) |

Supplementary table 1. *Continued*

| Variables | Use of beta blocker | Use of beta ACEi | Use of ARB | Use of statin |
| --- | --- | --- | --- | --- |
| Correlation Coefficient | *r* (*p*-value) | *r* (*p*-value) | *r* (*p*-value) | *r* (*p*-value) |
| Reactive hyperemia index (%) | -0.31  (0.77) | 0.17  (0.30) | -0.08  (0.43) | 0.14  (0.21) |
| Log Acetylcholine-induced iontophoresis  (ratio of response to baseline) | -0.26  (0.02) | 0.10  (0.35) | -0.16  (0.14) | -0.22  (0.40) |
| Log Nitropurusside-induced iontophoresis (ratio of response to baseline) | -0.13  (0.21) | -0.03  (0.75) | -0.09  (0.41) | 0.09  (0.47) |
